# Comparative Analysis of Health Economic Evaluations for Different Influenza Vaccines and Vaccination Strategies in China: A Systematic Review

**DOI:** 10.3390/vaccines13030332

**Published:** 2025-03-20

**Authors:** Fanxu Kong, Li Cai, Jiayi Zhang, Huijie Zhu, Zhibin Peng, Jiandong Zheng, Yaming Zheng, Hai Fang

**Affiliations:** 1 Chinese Field Epidemiology Training Program, Chinese Center for Disease Control and Prevention, Beijing 100050, China; kongfanxuczy@163.com; 2 Division of Infectious Disease, National Key Laboratory of Intelligent Tracking and Forecasting for Infectious Disease, Chinese Center for Disease Control and Prevention, Beijing 102206, China; 18665203507@163.com (L.C.); zjy15303414922@163.com (J.Z.); 17596539469@163.com (H.Z.); pengzb@chinacdc.cn (Z.P.); zhengjd@chinacdc.cn (J.Z.); 3 China Center for Health Development Studies, Peking University, Beijing 100191, China

**Keywords:** influenza vaccine, health economic evaluation, systematic review, China

## Abstract

Objective: This study systematically reviews health economic evaluations of influenza vaccines in China and synthesizes the evidence on different vaccine categories. Methods: We searched databases, including the China Hospital Knowledge Database, Wanfang, PubMed, Web of Science, and Embase, for studies on the health economics of influenza vaccines in China from 2015 to 2024. Studies were selected based on predefined criteria, and relevant data were extracted for analysis. The research utilized a parameter, ICER/threshold, defined as the ICER divided by the GDP per capita, to compare the results of cost-effectiveness analysis (CEA) studies. Results: A total of 1743 articles were identified, of which 25 met the inclusion criteria for full-text review. These included 19 Chinese studies and 6 English studies. Study populations were predominantly older adults (52.0%), followed by children, adolescents, people with chronic disease, and pregnant women. Vaccination strategies included trivalent inactivated influenza vaccine (TIV), quadrivalent inactivated influenza vaccine (QIV), trivalent live-attenuated influenza vaccine (LAIV), and non-vaccination groups. For TIV, 94.7% reported positive cost-effectiveness or cost-benefit results, with 21.1% identifying it as the most dominant strategy. For QIV, six studies compared it with a non-vaccinated group, and five (83.3%) reported favorable economic results. The study on LAIV showed cost-effectiveness compared to no vaccination, but not compared to QIV. The ICER threshold for TIV is the most favorable, and the population that exhibits the highest cost-effectiveness and benefit from vaccination is those people with underlying health conditions. Conclusions: TIV vaccination is often cost-effective, especially for people with chronic diseases, children, and older adults. Prioritizing TIV vaccination for those people with chronic diseases is recommended.

## 1. Introduction

Influenza is a prevalent acute respiratory disease to which the population is generally susceptible. Older people and those with immunocompromising underlying diseases tend to exhibit more severe symptoms following influenza infection. These groups also have the highest mortality rate from the disease [1]. Children and adolescents have the highest incidence rate of influenza [2]. In China, the influenza vaccine has been used for over two decades; however, the overall vaccination coverage in the entire population remains less than 5% [3], a figure that is substantially lower than that observed in economically developed countries in Europe as well as in the United States [4]. Some more highly developed regions of China have historically offered free influenza vaccine to older adults as well as primary and secondary school students with local household registration; nevertheless, the vaccination coverage rate remains low. For example, a study of hospitalized older adults in Beijing during the flu season from 2013 to 2019 revealed that the highest influenza vaccination coverage rate was lower than 20%, exhibiting a downward trend in recent years [5]. According to the most recent iteration of the China Influenza Vaccine Preventive Vaccination Technical Guidelines (2023–2024), vaccines authorized for use in China include three distinct types: the trivalent inactivated influenza vaccine (TIV), quadrivalent inactivated influenza vaccine (QIV), and trivalent live-attenuated influenza vaccine (LAIV).

Following introduction of the influenza vaccine in China, there has been a gradual increase in the number of studies involving the economic evaluation of influenza vaccines. However, the quality of existing studies remains unclear. Furthermore, there is currently no systematic assessment to determine which vaccine offers the best cost-effectiveness and which population group derives the greatest benefit from vaccination. The China Influenza Vaccine Preventive Vaccination Technical Guidelines (2023–2024) have only indicated that trivalent and quadrivalent vaccines are cost-effective, yet there is no definitive evidence regarding which vaccine is the most cost-effective. Therefore, this study aims to systematically analyze the economic evaluations of influenza vaccines published in China over the past decade, elucidating the current research status and quality. Furthermore, it will compare the effects and benefits of different vaccines available on the market to identify which vaccine offers optimal cost-effectiveness and in which population group the benefits are maximized. This analysis will provide compelling evidence to support the implementation of influenza vaccination policies.

## 2. Methods

This systematic review was prospectively registered in PROSPERO (ID: CRD420251006704) and implemented in rigorous compliance with the PRISMA (Preferred Reporting Items for Systematic Reviews and Meta-Analyses) reporting standards. Detailed methodological procedures are provided in Supplementary Materials Table S1.

### 2.1. Search Strategy

We followed a comprehensive strategy to systematically search the published literature for studies involving the health economic evaluation of influenza vaccines in China. We searched the China Hospital Knowledge Database, Wanfang, PubMed, Web of Science, and Embase databases. The search terms included subject terms and their free terms. The search formula used was as follows: influenza AND (vaccine OR vaccination) AND cost AND China. We referred to the temporal research scope outlined in the Cochrane Handbook for systematic reviews. Considering the relevance of influenza vaccine-related health economic research to current policy practices, we opted to analyze studies conducted over the past decade. The identified articles were then searched according to years of publication, which ranged from 1 January 2015 to 31 December 2024.

### 2.2. Inclusion and Exclusion Criteria

Inclusion criteria were as follows: (1) the study vaccine was an influenza vaccine; (2) the study area was mainland China; (3) the study methods were health economic evaluation methods, including cost-effectiveness analysis (CEA), cost–benefit analysis (CBA), and cost–utility analysis (CUA); and (4) the language of the publication was Chinese or English. The following were applied as the exclusion criteria: (1) the study was conducted in Hong Kong, Macao, or Taiwan, China; (2) combined vaccination with influenza and non-influenza vaccines; (3) the type of article was a review, conference, guidelines, or similar article; and (4) specific values for the outcome indicators were missing.

### 2.3. Literature Screening and Data Extraction

Following the identification of relevant literature, the title and abstract were downloaded and imported into NoteExpress. Two researchers independently reviewed article titles and abstracts according to the established inclusion and exclusion criteria. The full texts of articles deemed to meet these criteria were then read by both researchers to determine their eligibility for inclusion in this review. Disagreements were resolved by a third researcher. Data extraction was independently performed by two researchers, the extracted information mainly included basic information (author, publication year, and institution), study design (study year, target population, vaccine type, study area, and study perspective), data source (cost source and effect or benefit source), model information (model type, willingness-to-pay thresholds, and sensitivity analysis methods), and outcome indicators (benefit–cost ratio [BCR], net benefit, and incremental cost-effective ratio [ICER]). Furthermore, included studies varied in terms of research year, geographic region, cost data used, and the thresholds applied. These differences made comparison of results impossible. To address this issue, we proposed the ICER threshold ratio, defined as the ICER divided by the per capita Gross Domestic Product (GDP per capita) or per capita Gross Regional Product (GRP per capita). This ratio allowed us to overcome the difficulty of comparing results that stemmed from differences in research years and levels of economic development.

### 2.4. Literature Quality Evaluation

The included literature was evaluated using the Quality of Health Economic Studies (QHES) tool [6]. The QHES comprises 16 items, each of which is assigned a score ranging from 1 to 9 points according to weight, with a total score of 100 points. A score of >75 points indicates a high-quality study.

## 3. Results

### 3.1. Literature Screening

**Figure 1:**
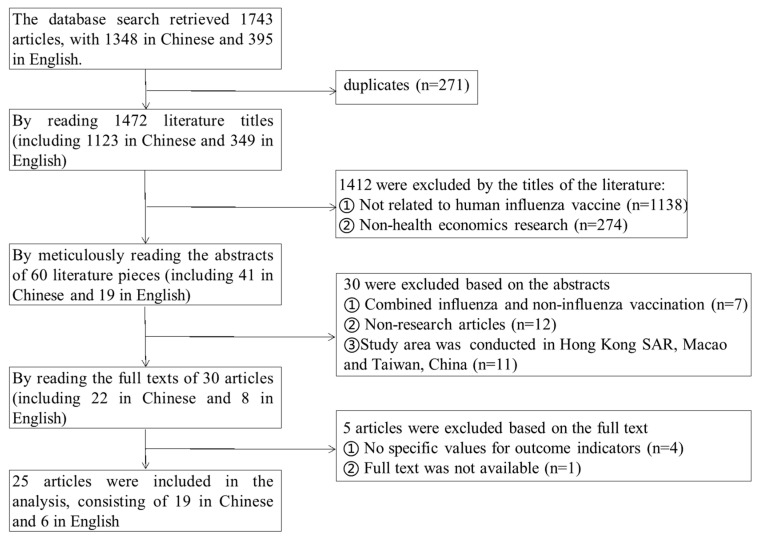
Flowchart of literature screening of health economics studies on influenza vaccination in China, 2015–2024.

Following a systematic search, a total of 1743 publications pertaining to the economic evaluation of influenza vaccines in China was obtained. Pursuant to application of the inclusion and exclusion criteria, 25 articles were ultimately included in this review [7,8,9,10,11,12,13,14,15,16,17,18,19,20,21,22,23,24,25,26,27,28,29,30,31], encompassing 19 articles in Chinese language and 6 articles in English. Figure 1 illustrates the process of literature screening.

### 3.2. Basic Characteristics of Included Studies

The landscape of health economic research on influenza vaccines in China has witnessed a progressive increase in the volume of publications, with the number of articles increasing from zero in 2017 to six in 2020 [15,16,24,25,26,27]. The entities engaged in this research encompassed academic institutions (n = 13, 52.0%), the Centers for Disease Control and Prevention (CDC) (n = 9, 36.0%), health care facilities (n = 2, 8.0%), and community-based organizations (n = 1, 4.0%). With respect to geographic distribution, the included studies spanned mainly four administrative tiers: national level (n = 8, 32.0%) [7,8,9,13,15,16,17,18], provincial level (n = 2, 8.0%, including directly controlled municipalities) [10,12], city level (n = 8, 32.0%) [11,23,24,25,27,29,30,31], and district level (n = 6, 24.0%) [14,19,20,21,22,26], with an additional publication at the level of the military (n = 1, 4.0%) [28]. Provinces with the highest representation in the study areas were Guangdong (n = 4, 16.0%) and Zhejiang (n = 3, 12.0%). Regarding the research perspectives adopted in the included studies, eight articles [9,10,11,12,13,15,16,18] adopted a societal perspective and two articles [7,17] focused on a health care perspective; the remaining publications did not explicitly describe their study perspective (Table 1).

### 3.3. Study Design

With regard to vaccine type, four vaccination schedules involving three influenza vaccines (TIV, QIV, and LAIV) as well as non-vaccination were examined, with TIV and QIV predominating. A total of 20 (80.0%) studies [7,8,9,10,11,14,15,17,18,19,20,21,22,23,24,26,28,29,30,31] analyzed trivalent inactivated vaccines, 8 (32.0%) studies [8,12,13,16,17,22,25,27] focused on quadrivalent inactivated vaccines, and 1 study [8] (4.0%) examined trivalent attenuated live vaccines. Regarding the settings of the intervention and control groups, there were a total of 15 (60.0%) studies [7,8,11,14,17,20,21,22,23,24,26,28,29,30,31] comparing the trivalent influenza vaccine with non-vaccination, 5 (20.0%) studies [9,10,15,18,19] examining government-paid TIV vs. self-paid TIV, 5 (20.0%) studies [8,17,22,25,27] comparing QIV with non-vaccination, 1 (4.0%) study [13] on government-paid QIV vs. non-vaccination, 3 (12.0%) studies [8,12,16] comparing QIV with TIV, 1 (4.0%) study [13] on government-paid QIV vs. government-paid TIV, and 1 (4.0%) study [8] analyzing LAIV vs. non-vaccination, TIV, or QIV. In terms of the vaccination rate settings, 16 studies [7,8,11,12,14,17,20,23,24,25,26,27,28,29,30,31] (64.0%) set the vaccination rate for intervention group at 100%, 2 studies [21,22] (8.0%) set it at 90%, and 7 studies [9,10,13,15,16,18,19] (28.0%) set it between 20% and 50%. For the control group, 22 studies [7,8,10,11,13,14,15,17,18,19,20,21,22,23,24,25,26,27,28,29,30,31] (88.0%) set the vaccination rate at 0%, while 5 studies [8,9,12,13,16] (20.0%) did not set it at 0%.

The targets for the studies comprised older people aged 60 years and above (n = 11, 44.0%) [11,13,14,15,16,17,19,20,24,25,30], children and adolescents (n = 6, 24.0%), pregnant women (n = 1, 4.0%), military recruits (n = 1, 4.0%), and other special groups (n = 2, 8.0%) [12,27], as well as people with underlying diseases (n =4, 16.0%) [7,18,26,31]. With regard to the origin of the parameters, in 10 (40.0%) studies, the costs were derived from surveys [14,20,23,25,26,27,28,29,30,31]; in 14 (56.0%), these data were from the literature or a public dataset [7,8,9,10,11,12,13,15,16,17,18,19,21,22]; and 1 study (4.0%) obtained cost data from both surveys and public datasets [24]. Additionally, 12 (92.3%) CEA studies involving quality-adjusted life year (QALY) data were sourced from the literature [7,8,9,10,11,12,13,15,16,17,18,19]; in 1 CEA study (7.7%), the data source was not described [14]. Among studies concerning CBA, 15.4% (n = 2) of the benefit data were from the literature [19,21], 46.2% (n = 6) from surveys [23,25,26,28,30,31], and 38.5% (n = 5) from surveys and publicly available data [20,22,24,27,29]. With regard to thresholds, eight studies (61.5%) used 1× GRP per capita as the threshold [7,9,10,11,13,15,18,19], and five studies (38.5%) used 3× GRP per capita as the threshold [8,12,14,16,17]. With regard to the evaluation methods used, 13 studies [7,8,9,10,11,12,13,14,15,16,17,18,19] (52.0%) used CEA, and 13 studies [19,20,21,22,23,24,25,26,27,28,29,30,31] (52.0%) conducted CBA; 1 study [19] (4.0%) performed both analyses. With regard to disease burden estimation, 10 (40.0%) studies used survey research, of which 7 [20,23,24,26,27,29,31] (70.0%) were prospective studies, and 3 [25,28,30] (30.0%) were retrospective. Fifteen (60.0%) studies used model evaluation, encompassing 13 (86.7%) static analytical models, including eight (52.0%) decision-tree models [9,12,13,15,16,18,19] and three (20.0%) Markov models [11,14,17]. One study each used a decision-tree–Markov model [10] (6.7%) and a decision-analytic model [8] (6.7%); two (13.3%) studies used dynamic analytic models [21,22], both susceptible–exposed–infected–recovered (SEIR) models. From the perspective of cost composition, 14 (56.0%) studies [8,9,12,15,16,22,23,24,25,26,27,28,29,31] included direct medical costs, direct non-medical costs, and indirect costs. Two (8.0%) studies [19,30] focused solely on direct medical costs. Additionally, two (8.0%) studies [14,17] incorporated both direct medical and direct non-medical costs, while five (20.0%) studies [10,11,13,18,20] included direct medical costs alongside indirect costs. Finally, two (8.0%) studies [7,21] did not clearly specify the costs involved. Concerning the sensitivity analyses, 12 (48%) studies [7,8,9,10,11,12,13,15,16,17,18,19] carried out the sensitivity analysis, 1 study [19] (8.3%) performed a one-way sensitivity analysis, 4 studies [12,13,15,18] (33.3%) conducted probabilistic sensitivity analysis, and 7 studies [7,8,9,10,11,16,17] (58.3%) carried out both one-way and probabilistic sensitivity analyses. The parameters exerting the most significant influence on the sensitivity analyses included the influenza incidence rate (4 studies, 50.0%) [9,10,11,17], the vaccine protection effect (2 studies, 25.0%) [8,16], the vaccine price (1 study, 12.5%) [19], and the severity of underlying diseases (1 study, 12.5%) [7].

### 3.4. Literature Quality Assessment

The literature of the included studies was assessed according to the QHES criteria; the results showed that 16 (64.0%) of the included studies had a quality score of >75, which indicates a high-quality study. The remaining nine studies (36.0%) included some with the highest quality score of 74 and some with the lowest quality score of 63, with a mean score of 71.7. The primary issues identified pertained to the absence of thorough elaboration of the study perspective (n = 15, 60.0%) or the rationale for selection of the perspective statement (n = 25, 100.0%), failure to adequately address uncertainty (n = 14, 56.0%), failure to articulate limitations (n = 7, 28.0%), and failure to explicitly discuss the direction and extent of potential bias in the study (n = 15, 60.0%) (Table S2 in Supplementary Materials).

### 3.5. Cost-Effectiveness Analysis

Thirteen studies [7,8,9,10,11,12,13,14,15,16,17,18,19] used the CEA method for vaccine health economic evaluation, which were categorized according to the selection of intervention and control groups; the results are listed below.

(a) TIV vs. non-vaccination

In five studies [7,8,11,14,17], the intervention group was vaccinated with TIV, and the control group was not vaccinated. Two studies (40.0%) showed that vaccination with TIV constitutes a highly advantageous strategy because it engenders an augmentation in QALYs concomitant with a reduction in total cost [8,11]. Three studies (60.0%) indicated that ICERs for TIV ranged from USD 346.4 to USD 9671.6 [7,14,17], falling within the cost-effectiveness threshold range.

With regard to ICER threshold ratios, these ranged from <0% to 99% across different study populations. The ranges of ICER threshold ratios for patients with chronic diseases [7], children and adolescents [8], and the elderly [11,14,17] were 2.7%, <0–8.8%, and <0–99.0%, respectively (Table 2).

(b) Government-funded TIV vs. self-funded TIV

Five studies compared an intervention group, in which individuals were vaccinated with government-funded TIV, and a control group, in which participants incurred the cost of vaccination with self-funded TIV [9,10,15,18,19]. Most of these studies used 2012 Beijing vaccination data for older adults and the local economic level as reference [32]; in the control group, the vaccination rate varied from 0% to 6.95%. The control group’s vaccination rate was either based on the actual local vaccination rate or was designated as non-vaccination. Two studies [10,19] (40.0%) demonstrated that government-funded TIV vaccination was the optimal strategy for achieving a greater number of QALYs and reducing the total cost of TIV vaccination, as compared with self-funded vaccination. Furthermore, an increase in the vaccination rate was associated with a decrease in total cost and decrease in QALY loss. Conversely, three studies [9,15,18] (60.0%) reported cost-effective ICERs ranging from USD 1519.3 to USD 7964 in the government-funded vaccination group compared with self-funded vaccination, all falling within the threshold.

With regard to ICER threshold ratios, these varied from less than 0% to 78.5% across different populations. The ranges of ICER threshold ratios for patients with chronic diseases [18], children and adolescents [9], and the elderly [15,19] were 18.8%, 78.5%, and <0–54.7% respectively (Table 2).

(c) QIV vs. non-vaccination

In three studies [8,13,17], the intervention group was vaccinated with QIV, and the control group was not vaccinated. The study areas were nationwide in all studies. Two of these studies [8,17] (66.7%) involved QIV in the intervention group and non-vaccination in the control group, with ICERs of USD 7703.5 and USD 26,296.2, respectively, which were both within the 3× GRP per capita threshold. The cost-effectiveness of QIV is illustrated by the findings of these two studies. In contrast, another study [13] (33.3%) reported QIV vaccination in the intervention group and non-vaccination in the control group, with an ICER value of USD 10,916.7, which was slightly above the 1× GRP per capita threshold and did not demonstrate a cost-effectiveness effect. ICER threshold ratios across different populations ranged from 61.4% to 269.3%. The ranges of ICER threshold ratios for children and adolescents [8] and the elderly [13,17] were 61.4% and 106.3–269.3%, respectively (Table 2).

(d) QIV vs. TIV

In four studies [8,12,13,16], QIV in the vaccine group was compared with TIV in the control group, with the same vaccination rate. Two studies [8,12] (50.0%) reported a 100% vaccination rate; the other two studies (50.0%) reported vaccination rates of 47.5% [13] and 26.7% [16]. Three studies [8,12,16] (75.0%) demonstrated that the ICER for QIV vaccination ranged from USD 6700 to USD 32,948.5, all falling within the threshold and indicating cost-effectiveness. Conversely, one study [13] (25.0%) revealed an ICER of USD 123,550.7 for QIV vaccination, which exceeded the threshold and was not considered cost-effective. ICER threshold ratios varied between 57.1% and 262.5% across different populations. The ranges of ICER threshold ratios for children and adolescents [8] and the elderly [13,16] were 262.5% and 70.0–1202.5%, respectively (Table 2).

(e) LAIV vs. QIV/non-vaccination

One study [8] involved an intervention group that was fully vaccinated with LAIV, in comparison to a group that did not receive any vaccination or was fully vaccinated with QIV. Compared with the non-vaccination group, the ICER for LAIV vaccination was USD 24,739.1, which was cost-effective and within the threshold. Conversely, when LAIV was compared with QIV vaccination, the ICER was USD 123,983.8, which was not cost-effective and above the threshold. Preliminary research suggests that the use of LAIV is cost-effective when the protective rate exceeds 79%, or when the cost is reduced to USD 28.22 (with QIV costing USD 18.29). It should be noted that no comparative analysis of LAIV vs. TIV was performed in that past study. The ICER threshold ratios for LAIV vs. non-vaccination and LAIV vs. QIV were 197.1% and 987.8%, respectively.

### 3.6. Cost–Benefit Analysis

In 13 studies [19,20,21,22,23,24,25,26,27,28,29,30,31], economic evaluations of vaccines were conducted using the CBA method, with BCR serving as the outcome indicator. The results are reported according to the choice of intervention and control groups, as below (Table 3).

(a) TIV vs. non-vaccination

Eleven studies examined TIV vaccination in the intervention group and non-vaccination in the control group; in eight studies (72.7%), participants in the intervention group were fully vaccinated. Among studies with TIV in the intervention group [20,23,24,26,28,29,30,31], three (27.3%) included partially vaccinated (90%) participants [19,21,22]. Statistical analysis revealed that the cost of TIV vaccination ranged from USD 4.5 to USD 14.96, the benefit of vaccination ranged from USD 7.2 to USD 414.9, and the per capita benefit ranged from USD −1.5 to USD 406. After administration of the TIV, all studies but one [29] (90.9%) demonstrated cost benefits, with BCRs ranging from 0.83 to 48.7, with a median of 5.72. With regard to the timing of vaccination, studies such as that by Xiuyun Chen et al. [29] demonstrated that the BCRs for children in nursery school were 0.28, 0.85, and 0.83, which were lower than 1; there were no cost benefits at 4, 6, and 8 months after TIV vaccination. However, a study among older adults receiving TIV yielded contrary results [20], with the BCR increasing gradually at 1, 3, and 6 months post-vaccination at ratios of 1.4, 9.7, and 13.1, respectively, indicating a significant benefit from vaccination. In terms of vaccination coverage, Wu Jiajing et al. [19] demonstrated that the BCR value remained relatively stable when the vaccination rate in the vaccine group increased gradually. In contrast, Yucheng Xu et al. [21] showed that the BCR value increased gradually with an increased vaccination rate.

In terms of the population, the benefit–cost ratio ranges for vaccination among patients with chronic diseases [26,31], children and adolescents [21,22,23,29], as well as the elderly over 60 years old [19,20,24,30], were 10.09–48.67%, 0.83–9.97%, and 1.26–13.06%, respectively. Older patients with chronic obstructive pulmonary disease [31] achieved the highest BCR (48.67) after receiving TIV, indicating that the influenza vaccine has an important role in reducing the economic burden for individuals with underlying respiratory diseases (Table 3).

(b) QIV vs. non-vaccination

A total of three studies [22,25,27] examined QIV vaccination in the intervention group and non-vaccination in the control group. Of these, participants in the intervention group of two studies [25,27] (66.7%) were fully vaccinated, and those in the intervention group of the third study [22] (33.3%) were partially vaccinated. The BCRs for QIV vaccination ranged from 2.4 to 4.1, all of which were cost-beneficial. As demonstrated by Xiang et al. [22], the cost of vaccination is a crucial indicator of its benefits. Whereas QIV vaccination can prevent more cases of influenza, its price is much higher than that of TIV, and the resulting BCR value is actually lower. In terms of the population, the benefit–cost ratio ranges for vaccination in preschool children and the elderly are 4.10 and 2.42.

## 4. Discussion

In this review, we conducted a systematic search of major databases for studies in both Chinese and English language on the economics of three types of influenza vaccines that have been approved in China. A preliminary description of differences in the effectiveness and benefits of vaccination in different populations was also provided. Of the 25 studies included in this review, most described the cost-effectiveness of influenza vaccination or increased vaccination coverage rates. Of the included studies, 19 focused on TIV vaccination, 18 of which found that TIV was cost-effective or cost-beneficial in three or more comparisons with the non-vaccination group. Of the QIV vaccination studies, six found QIV to be cost-effective or cost-beneficial in five comparisons with the non-vaccination group, and four studies showed it to be cost-beneficial in three comparisons with TIV. In contrast, only one study found LAIV vaccination to be cost-effective when compared with the non-vaccination group. Our study findings generally align with those of a systematic review on influenza vaccination in Europe and the United States by D’Angiolella et al. [33], who reported that influenza vaccination is typically cost-effective across various population subgroups. Consequently, it is imperative to enhance the influenza vaccination coverage rate within the population to minimize the detrimental effect on population health and reduce the associated economic burden.

Our review revealed that several studies exhibited deficiencies with regard to standardization and criteria consistency. Firstly, the QHES standard score is relatively low, indicating that many current studies have not referred to guidelines of economic evaluation for vaccines. Secondly, the included studies demonstrate significant differences in cost composition, which can lead to substantial variations in the results across different studies. Thirdly, there are notable discrepancies in the methods used for estimating disease burden. The estimation of disease burden directly influences evaluation outcomes and contributes to heterogeneity among studies. Fourthly, the setting of vaccination rates, such as establishing it at 100%, does not reflect real-world scenarios. Fifth, in selecting model parameters, the absence of local data prompted some studies to depend on foreign data as a substitute, thereby raising concerns about the reliability of the results. It is therefore recommended that researchers refer to the quality checklist for economic evaluations of vaccines when conducting research, so as to standardize the research design. It is also encouraged to use dynamic models for estimating the disease burden [34]. Prioritizing the use of the most recent data from the local area or adjacent regions exhibiting comparable conditions is strongly advised. In instances where such data are not available, systematic reviews or meta-analyses can be used as a means of estimating outcomes, thereby ensuring optimal accuracy and facilitating the comparison of studies.

Many studies have identified discrepancies in economic evaluations of various influenza vaccinations, with TIV vaccination demonstrating the highest cost-effectiveness and cost benefits in CEA studies. When the control group was not vaccinated, the ICER threshold ratios for the intervention group vaccinated with TIV ranged from negative to 99.0%, which were lower than those for vaccination with QIV and LAIV, which ranged from 61.4% to 269.3%. In CBA studies, the control group was not vaccinated, and the median and maximum BCRs for TIV vaccination in the vaccine group were 5.7 and 48.7, respectively, which were higher than those for QIV vaccination, which were 2.7 and 4.1, respectively. Hendriks et al. [35] suggested that, given the markedly elevated cost of QIV relative to TIV in low- and middle-income countries, the strategy of administering TIV to a greater number of individuals can generate more substantial health benefits. In high-income countries such as the United States, where the government has greater fiscal capacity, the additional cost of QIV compared with TIV is relatively minor (an increase of USD 2–5) [36], making it more probable that QIV is cost-effective [37]. It is important to note that in recent years, no confirmed cases of influenza caused by the B/Yamagata lineage wild strain have been detected in global surveillance. Consequently, the World Health Organization has advocated a systematic transition from QIV to TIV, a shift that the United States has already initiated by reverting to use of the TIV for the entirety of the 2024/2025 flu season. In light of the prevailing influenza virus circulation lineages and budgetary constraints, prioritizing TIV vaccination is recommended.

Research has identified specific patterns in the net benefits of flu vaccination across diverse populations. A comparison of the per capita vaccination benefits of TIV has revealed that individuals with chronic diseases [26,31] exhibit the most significant benefits, particularly older patients with chronic respiratory diseases. Younger populations [21,22,23] also demonstrate notable benefits, with young children exhibiting a greater benefit. Furthermore, healthy older individuals [19,20,24,25,30] and the general population [28] exhibit substantial benefits. A similar pattern was observed in two studies on QIV vaccination [22,25]. The net benefit of vaccination for preschool children was found to be USD 121.0, which is higher than the USD 24.3 for the older population without chronic diseases. A study of four European countries also indicated that older adults and individuals with chronic diseases are priority groups for influenza vaccination, with the aim of maximizing public health and economic benefits [38]. This finding underscores the importance of prioritizing these key groups in recommending and promoting vaccination during the influenza season.

This study has several limitations. First, the uneven quality of the included studies and certain methodological flaws may compromise the reliability and validity of the results. Second, this study exclusively considered published documents, omitting some unpublished research reports, which could lead to an overestimation of the intervention’s effect and introduce bias into the findings. Finally, due to the limited number of the included literature and their significant heterogeneity, only a systematic review was performed, and a meta-analysis was not feasible. Consequently, the findings are presented in a qualitative manner, lacking the precision that quantitative analysis could provide.

## 5. Conclusions

In summary, although the growing body of research on influenza vaccine economic evaluations has expanded in recent years, their methodological rigor remains suboptimal. Future studies need to be conducted in accordance with guidelines for vaccine economic evaluations, while incorporating standardized metrics that better reflect real-world transmission dynamics. Our findings demonstrate that trivalent inactivated influenza vaccines (TIV) exhibited the most favorable cost-effectiveness profile, with chronic disease patients achieving the highest health gains across all subgroups. This evidence supports policy-makers in resource-sufficient settings allocating TIV preferentially to this high-risk population.

## Figures and Tables

**Table 1:** Summary of basic information from health economics research studies on influenza vaccination in China, 2015–2024.

First Author	Publication Year	Author Affiliation	Region	Research Perspective	Study Year	Population	Intervention Group	Vaccination Rate in the Intervention Group (%)	Control Group	Vaccination Rate in the Control Group (%)	Assessment Type	Cost Data Source	Cost Composition	Effectiveness or Benefit Data Source	Model	Sensitivity Analysis
Minting Zhao [7]	2024	College	China	Health care	2022	Heart failure patients	TIV	100	NV	0	CEA	Public dataset	NES	Literature	MM	One-way, PSA
Yilin Gong [8]	2023	College	China	Society	2021	6–35 months	TIV	100	NV	0	CEA	Literature and public dataset	dmc, dnmc, ic	Literature	DAM	One-way, PSA
						3–18 years	TIV	100	NV	0						
							QIV	100	NV	0						
							LAIV	100	NV	0						
							QIV	100	TIV	100						
							LAIV	100	QIV	100						
Qiang Wang [9]	2023	College	China	Society	2019	<14 years	gfTIV	40	sfTIV	6.95	CEA	Literature	dmc, dnmc, ic	Literature	DTM	One-way, PSA
Jiaxin Wen [10]	2023	CDC	Jiangsu	Society	2022	Pregnant women	gfTIV	30	sfTIV	0	CEA	Literature and public dataset	dmc, ic	Literature	DT-MM	One-way, PSA
Xiaoliang Wu [11]	2022	CDC	Guangdong	Society	2019	>60 years	TIV	100	NV	0	CEA	Public dataset	dmc, ic	Literature	MM	One-way, PSA
Dawei Zhu [12]	2022	College	Beijing	Society	2019	At-risk population	QIV	100	TIV	100	CEA	Literature and public dataset	dmc, dnmc, ic	Literature	DTM	PSA
Han Yan [13]	2021	College	China	Society	2019	>60 years	gfQIV	47.5	NV	0	CEA	Literature and public dataset	dmc, ic	Literature	DTM	PSA
									gfTIV	47.5						
Yan Luo [14]	2021	Community	Sichuan	NES	2019	>63 years	TIV	100	NV	0	CEA	Investigation	dmc, dnmc	Investigation	MM	NC
Juan Yang [15]	2020	College	China	Society	2017	>60 years	gfTIV	30	sfTIV	0	CEA	Literature and public dataset	dmc, dnmc, ic	Literature	DTM	PSA
Minghuan Jiang [16]	2020	College	China	Society	2019	>60 years	QIV	26.7	TIV	26.7	CEA	Literature and public dataset	dmc, dnmc, ic	Literature	DTM	One-way, PSA
Chen Chen [17]	2019	College	China	Health care	2018	>60 years	QIV	100	NV	0	CEA	Literature and public dataset	dmc, dnmc	Literature	MM	One-way, PSA
							TIV	100	NV	0						
Juan Yang [18]	2019	College	China	Society	2016	Diabeticpatients	gfTIV	40	sfTIV	0	CEA	Literature and public dataset	dmc, ic	Literature	DTM	PSA
Jiajing Wu [19]	2018	Hospital	Zhejiang	NES	2017	>65 years	gfTIV	20	sfTIV	0	CEA, CBA	Literature	dmc	Literature	DTM	One-way
Pufang Li [20]	2024	CDC	Hunan	NES	2021–2022	>65 years	TIV	100	NV	0	CBA	Investigation	dmc, ic	Investigation and public dataset	NC	NC
Yucheng Xu [21]	2022	CDC	Guangdong	NES	2016–2019	Children and adolescents	TIV	90	NV	0	CBA	Literature	NES	Literature	SEIR	NES
Yingfei Xiang [22]	2021	College	Guangdong	NES	2019	Children and adolescents	TIV	90	NV	0	CBA	Public dataset	dmc, dnmc, ic	Investigation and public dataset	SEIR	NES-
							QIV	90	NV	0						
Chenlu He [23]	2021	Hospital	Qinghai	NES	2019–2020	Children and adolescents	TIV	100	NV	0	CBA	Investigation	dmc, dnmc, ic	Investigation	NC	NC
Nianchu Liu [24]	2020	CDC	Zhejiang	NES	2018	>60 years	TIV	100	NV	0	CBA	Investigation and public dataset	dmc, dnmc, ic	Investigation and public dataset	NC	NC
Yan Wang [25]	2020	College	Shandong	NES	2019	>70 years	QIV	100	NV	0	CBA	Investigation	dmc, dnmc, ic	Investigation	NC	NC
Minrui Xu [26]	2020	CDC	Jiangsu	NES	2019	Patients with chronic disease	TIV	100	NV	0	CBA	Investigation	dmc, dnmc, ic	Investigation	NC	NC
Yating Wu [27]	2020	College	Henan	NES	2018–2019	Patients aged >60 years with unstable angina	QIV	100	NV	0	CBA	Investigation	dmc, dnmc, ic	Investigation and public data	NC	NC
Dongqi Gao [28]	2016	CDC	Troup	NES	2014	New soldiers aged 18–20 years	TIV	100	NV	0	CBA	Investigation	dmc, dnmc, ic	Investigation	NC	NC
Xiuyun Chen [29]	2016	CDC	Guangdong	NES	2017	Children in nursery	TIV	100	NV	0	CBA	Investigation	dmc, dnmc, ic	Investigation and public dataset	NC	NC
Ping Zhang [30]	2015	CDC	Shanxi	NES	2013–2014	>65 years	TIV	100	NV	0	CBA	Investigation	dmc	Investigation	NC	NC
Bijun Shi [31]	2015	College	Zhejiang	NES	2013	>60 years COPD patient	TIV	100	NV	0	CBA	Investigation	dmc, dnmc, ic	Investigation	NC	NC

NES, not explicitly stated; NC, not conducted; DTM, decision-tree model; MM, Markov model; DT-MM, decision-tree–Markov model; DAM, decision-analytic model; SEIR, susceptible–exposed–infected–recovered model; PSA, probabilistic sensitivity analysis; One-way, one-way sensitivity analysis; gfTIV, government-funded TIV; sfTIV, self-funded TIV; NV, non-vaccination; dmc, direct medcial cost; dnmc, direct non-medical cost; ic, indirect cost.

**Table 2:** Studies on the cost-effectiveness of influenza vaccination in China, 2015–2024.

Reference Number	Target Population	Intervention Group	Control Group	Threshold	ICER	ICER/ 1 GDP (GRP) PC (%)
[7]	Heart failure patients	TIV	NV	1 GDP PC:12733.7 USD	346.4 USD	2.7
[8]	6–35 months	TIV	NV	3 GDP PC:37653 USD	<0	<0
	3–18 years	TIV	NV		1102.5 USD	8.8
		QIV	NV		7703.5 USD	61.4
		LAIV	NV		24,739.1 USD	197.1
		QIV	TIV		32,948.5 USD	262.5
		LAIV	QIV		123,983.8 USD	987.8
[11]	>60 years	TIV	NV	1 GDP PC:10274.2 USD	<0	<0
[14]	>63 years	TIV	NV	3 GDP PC:28106.1 USD	9356 USD	97.7
[17]	>60 years	TIV	NV	3 GDP PC:29294.9 USD	9671.6 USD	99.0
		QIV	NV		26,296.2 USD	269.3
[9]	<14 years	gfTIV	sfTIV	1 GDP PC:10144 USD	7964 USD	78.5
[10]	Pregnant women	gfTIV	sfTIV	1 GDP PC:21454.7 USD	<0	<0
[15]	>60 years	gfTIV	sfTIV	1 GDP PC:8840 USD	4832 USD	54.7
[18]	Patients with diabetes	gfTIV	sfTIV	1 GDP PC:8084.3 USD	1519.3 USD	18.8
[19]	>65 years	gfTIV	sfTIV	1 GDP PC:15955.6 USD	<0	<0
[13]	>60years	gfQIV	NV	1 GDP PC:10274.2 USD	10,916.7 USD	106.3
			gfTIV		123,550.7 USD	1202.5
[12]	At-risk population	QIV	TIV	3 GDP PC:71338 USD	13,580 USD	57.1
[16]	>60years	QIV	TIV	3 GDP PC:29580 USD	6700 USD	70.0

gfTIV, government-funded TIV; sfTIV, self-funded TIV; NV, non-vaccination. ICER < 0 indicates that, compared to the control strategy, the intervention group had less cost and higher effectiveness.

**Table 3:** Studies on the cost benefits of influenza vaccination in China, 2015–2024.

Reference Number	Target Population	Intervention Group	Control Group	Net Benefits per Capita/USD	Benefit–Cost Ratio
[20]	>65 years	TIV	NV	169.4	13.06
[23]	Children and adolescents	TIV	NV	87.1	9.97
[24]	>60 years	TIV	NV	24.7	5.61
[26]	Patients with chronic disease	TIV	NV	882.5	10.09
[28]	New soldiers aged 18–20 years	TIV	NV	30.7	5.44
[29]	Children in nursery school	TIV	NV	−1.5	0.83
[30]	>65 years	TIV	NV	13.9	3.80
[31]	Patients aged >60 years with COPD	TIV	NV	406.4	48.67
[21]	Children and adolescents	TIV	NV	83.4	7.88
[19]	>65 years	gfTIV	sfTIV	0.46	1.26
[22]	Preschool children	TIV	NV	79.6	7.24
		QIV	NV	122.7	4.10
[25]	>70 years	QIV	NV	24.7	2.42
[27]	Patients aged >60 years with unstable angina	QIV	NV	39.4	2.77

gfTIV, government-funded TIV; sfTIV, self-funded TIV; NV, non-vaccination.

## Data Availability

The original contributions presented in this study are included in the article. Further inquiries can be directed to the corresponding author.

## Supplementary Materials

The following supporting information can be downloaded at: https://www.mdpi.com/article/10.3390/vaccines13030332/s1, Table S1. The PRISMA 2020 statement. Table S2. Quality evaluation of health economics studies on influenza vaccination in China, 2015–2024. Reference [39] are cited in the supplementary materials.
